# Neural bases of accented speech perception

**DOI:** 10.3389/fnhum.2015.00558

**Published:** 2015-10-06

**Authors:** Patti Adank, Helen E. Nuttall, Briony Banks, Daniel Kennedy-Higgins

**Affiliations:** ^1^Division of Psychology and Language Sciences, Department of Speech, Hearing, and Phonetic Sciences, University College LondonLondon, UK; ^2^School of Psychological Sciences, University of ManchesterManchester, UK

**Keywords:** cognitive neuroscience, speech perception, accented speech, fMRI, speech in noise, noise-vocoded speech, time-compressed speech

## Abstract

The recognition of unfamiliar regional and foreign accents represents a challenging task for the speech perception system (Floccia et al., 2006; Adank et al., 2009). Despite the frequency with which we encounter such accents, the neural mechanisms supporting successful perception of accented speech are poorly understood. Nonetheless, candidate neural substrates involved in processing speech in challenging listening conditions, including accented speech, are beginning to be identified. This review will outline neural bases associated with perception of accented speech in the light of current models of speech perception, and compare these data to brain areas associated with processing other speech distortions. We will subsequently evaluate competing models of speech processing with regards to neural processing of accented speech. See Cristia et al. (2012) for an in-depth overview of *behavioral* aspects of accent processing.

## Processing accent variation at pre- and post-lexical levels

Models outlining the neural organization of speech perception (Hickok and Poeppel, 2007; Rauschecker and Scott, 2009) propose that the locus of processing intelligible speech is the temporal lobe within the ventral stream of speech processing. Rauschecker & Scott suggest that intelligibility processing has its center of gravity in left anterior STS (Superior Temporal Sulcus), while Hickok & Poeppel propose that processing intelligible speech is bilaterally organized and located both anteriorly and posteriorly to Heschl's Gyrus. However, both models are based on intelligible speech perception and do not make explicit predictions about the cortical substrates that subserve speech perception under challenging listening conditions (cf. Adank, 2012a) for a discussion on processing of intelligible speech).

A handful of fMRI studies address how the brain processes accent variation. Listening to difficult foreign phonemic contrasts (e.g., /l/-/r/ contrasts for Japanese listeners) has been associated with increased activation in auditory processing/speech production areas, including left Inferior Frontal Gyrus (IFG), left insula, bilateral ventral Premotor Cortex, right Pre- and Post-Central Gyrus, left anterior Superior Temporal Sulcus and Gyrus (STS/STG), left Planum Temporale (PT), left superior temporal parietal area (Stp), left Supramarginal Gyrus (SMG), and cerebellum bilaterally (Callan et al., 2004, 2014). It is noteworthy that the neural bases associated with listening to foreign languages overlap with those reported for unfamiliar accent processing, including bilateral STG/STS/MTG, and left IFG (Perani et al., 1996; Perani and Abutalebi, 2005; Hesling et al., 2012).

**Figure 1:**
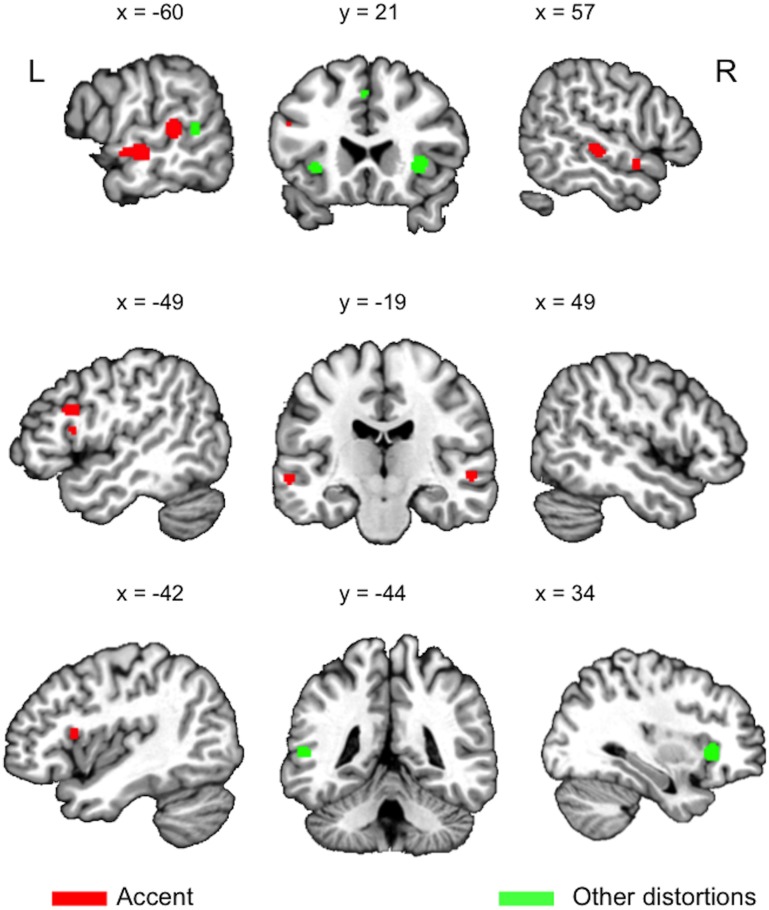
**Clusters (logical) resulting from an Activation Likelihood Estimation (ALE) analysis conducted using GingerALE 2.3.3 (www.brainmap.org), *q* < 0.0001, cluster extent of 100 mm^3^, for the four *accent* studies (red), and the seven *other distortions* studies (pooled noise, time-compressed, and noise-vocoded studies) (green)**.

For sentence processing (Table 1, Figure 1), listening to an unfamiliar accent involves a network of frontal (left IFG, both Operculi/Insulas, Superior Frontal Gyrus), temporal (left Middle Temporal Gyrus [MTG], right STG), and medial regions (Supplementary Motor Area [SMA]) (Adank, 2012b; Adank et al., 2012b, 2013; Yi et al., 2014). It is unclear how the accent processing network maps onto the networks in Rauschecker and Scott (2009) and Hickok and Poeppel (2007). The coordinates for accent processing in the left temporal lobe are located anteriorly and posteriorly to Hickok and Poeppel's proposed STG area for spectrotemporal analysis, while the coordinates in left IFG are located inside Hickok and Poeppel's left inferior frontal area assigned to the dorsal stream's articulatory network. In contrast, the temporal coordinates in Table 1 fit well with Rauschecker & Scott's antero-ventral and postero-dorsal areas placed anteriorly and posteriorly to left primary auditory cortex, respectively, and the left IFG coordinates fall within their antero-ventral left inferior frontal area.

**Table 1:** **Reported brain regions in studies investigating processing of accented, time-compressed, or noise-vocoded speech, plus speech with added background noise vs. undistorted words or sentences**.

**Distortion**	**Study**	**Contrast**	**MNI**	**Location**	Original location^*^
Unfamiliar accent	Adank et al., 2012b	Sentences unfamiliar > sentences familiar accent	−54, −40, 4	L MTG	L Post. STG/SMG
			−60, −34, 8	L MTG	L Post. STG/PT
			−60, −26, −4	L MTG	L Post. MTG
			60, −32, 2	R STG	R Post. STG/SMG
			−50, 12, 24	L POp	L POp/PG
			−46, 16, 12	L POp	L POp/PTr
			54, −26, −2	R STG	R Post. STG/MTG/SMG
			54, 4, −16	R RO	R Ant. STG/TP/MTG
			38, 18, 26	R PTr	R Central Opercular Cortex
	Adank et al., 2012a	Sentences in unfamiliar > sentences in familiar accent	−60, −12, −6	L MTG	L STG/STS
	Adank et al., 2013	Sentences in unfamiliar accent > unintelligible sentences	−62, −32, 4	L MTG	L STS
			−58, −4, −8	L FO	L STG
			−60, −16, −8	L MTG	L MTG
			−50, 18, 24	L PTr	L IFG PTr)
			−46, 28, −4	L POrb	L IFG POrb)
			−36, 22, −4	L Insula	L Insula
			56, −20, −6	R STG	R STG
			60, 2, −12	R STG	R STG
			−2, 10, 60	L SMA	L SMA
	Yi et al., 2014	Sentences in foreign accent > sentences in native accent	4, 24, 34	R MCC	R Paracingulate Gyrus
			34, −52, 62	R SPL	R Motor cortex, SPL, somatosensory cortex
			−40, 14, 8	L Insula	L Insula
			20, −2, 60	R SFG	R SFG
			32, 20, −6	*No location given*	R Insula
			−52, 10, 10	L POp	L IFG
			−26, 24, 0	L Insula	L Insula
			42, 14, 8	R IFG	R Insula
Time−compressed speech	Adank and Devlin, 2010	Time−compressed > normal−speed sentences	−60, −14, 0	L MTG	L Ant. STG/STS
			−58, −46, 4	L MTG	L Post. STG/STS
			64, −14, 0	R STG	R Ant. STG/STS
			56, −32, 4	R STG	R Post. STG/STS
			0, 12, 60	SMA	Pre−SMA
			0, 22, 44	SMA	Cingulate sulcus
			−36, 24, −4	L Insula	L FO
			36, 25, 2	R Insula	R FO
	Peelle et al., 2004	Time−compressed > normal−speed sentences	−28.38, −66.82, 47.33	L SPL	L Posterior parietal BA19/39/40)
			−28.54, −76.78, 32.63	L MOG	L Inferior parietal BA19/39)
			−54.12, −38.58, −16.66	L STG	L Inferior temporal BA20)
			−15.43, −62.52, 46.69	L SPL	L Posterior parietal BA7)
			14.07, −23.17, −4.77	R Thalamus	R Thalamus
			13.99, −7.32, −7.46	R Thalamus	R Subthalamic nucleus
			1.02, −38.08, −14.28	R Cerebellar Vermis	R Cerebellum
	Poldrack et al., 2001	Compression−related increases during sentence processing	−28, 54, 16	L MFG	L MFG
			34, 26, −4	R Insula	R IFG/Insula
			4, 32, 20	R ACC	R ACC
			18, 4, 8	*No location given*	Striatum
			66, −40, 8	R MTG	R STG
Noise−vocoded speech	Erb et al., 2013	Noise−vocoded > clear sentences	−6, 26, 40	L SMedG	L SMA/ACC
			−30, 20, −5	L Insula	L Ant. Insula
			33, 23, −3	R Insula	R Ant. Insula
			−9, 11, 7	L Caudate Nucleus	L Caudate Nucleus
			12, 17, 10	R Caudate Nucleus	R Caudate Nucleus
	Zekveld et al., 2014	Noise−vocoded > clear sentences	−4, 8, 60	L SMeDG	L SFG
			−64, −40, 10	L STG	L STG
			−48, −42, 2	L MTG	L MTG
			−44, −38, 8	L STG	L MTG
Background noise	Adank et al., 2012a	Sentences in background noise > sentences in quiet	32, 28, 10	*No location given*	R IFG/FO
			−32, 24, 8	L Insula	L FO/IFG/Insula
			6, 14, 28	*No location given*	R Cingulate Gyrus
			−24, 40, −2	*No location given*	L Parahippocampal Gyrus
			−12, 10, −2	L Putamen	L Caudate
			12, 20, 36	R MCC	R Paracingulate/Cingulate
			30, 40, 24	R MFG	R Frontal Pole
			8, 22, 18	*No location given*	R Cingulate Gyrus
	Peelle et al., 2010	Sentences in continuous scanning EPI Sequence > sentences in quiet EPI sequence	−36, −74, 44	L IPL	L Inferior parietal cortex
			−40, −66, 44	L AG	L Angular gyrus
			−48, −60, 48	L IPL	L Inferior parietal cortex
			−56, −46, 8	L MTG	L Post. MTG
			−66, −44, 0	L MTG	L Post MTG
			−68, −14, 2	L STG	L Ant. STS
			−68, 2, −8	*No location given*	L Ant. STS
			−60, 4, −14	L STG	L Ant. STS

Note that the list of papers is not exhaustive. Coordinates in Talairach space were converted to MNI space using the tal2icbm_spm algorithm www.brainmap.org/ale. Anatomical locations determined using the Anatomy ToolBox (Eickhoff et al., 2005, 2006, 2007) in SPM8 Wellcome Imaging Department, University College London, London, UK).

* *Original location as reported in the study. AG, Angular Gyrus; FFG, Fusiform Gyrus; FO, Frontal Operculum; IFG, Inferior Frontal Gyrus; IOG, Inferior Occipital Gyrus; IPL, Inferior Parietal Lobule; MCC, Middle Cingulate Cortex; MFG, Middle Frontal Gyrus; MTG, Middle Temporal Gyrus; PG, Precentral Gyrus; POp, Pars Opercularis; PT, Planum Temporale; PTr, Pars Triangularis; POrb, Par Orbitalis: RO, Rolandic Operculum; SMA, Supplementary Motor Area; SMedG, Superior Medial Gyrus; SMG, Supramarginal Gyrus; STG, Superior Temporal Gyrus; STG, Superior Temporal Planum; STS, Superior Temporal Sulcus; TP, Temporal Pole*.

## Accented speech vs. other challenging listening conditions

As is the case with other types of distorted speech, understanding accented speech is associated with increased listening effort (Van Engen and Peelle, 2014). However, accent variation is of a conceptually different nature than variation in the acoustic signal resulting from an extrinsic source such as noise, i.e., phonetic realizations that differ from the listener's native realization of speech sounds. Furthermore, in contrast to speech-intrinsic variation, noise compromises the auditory system's representation of speech from ear to brain. Accented speech also differs from distortions such as noise-vocoded or time-compressed speech as the variation does not affect the acoustic integrity of the acoustic signal, as only specific phonemic and suprasegmental characteristics vary.

Processing speech in noise involves areas also activated for speech in an unfamiliar accent (Table 1): left insula (Adank et al., 2012a), left MTG (Peelle et al., 2010), left Pars Opercularis (POp), bilateral Pars Triangularis (PTr). Comprehension of time-compressed sentences activates left MTG (Poldrack et al., 2001; Adank and Devlin, 2010), right STG (Peelle et al., 2004; Adank and Devlin, 2010), SMA and left Insula (Adank and Devlin, 2010), while noise-vocoded speech activates left Insula (Erb et al., 2013), and left MTG/STG (Zekveld et al., 2014). However, it is clear from Figure 1 that processing accented speech also activates areas outside the network activated for processing speech in noise, time-compressed speech, and noise-vocoded speech.

Another problem in identifying networks governing accent processing is that perceiving variation in an unfamiliar accent (i.e., in an accent that differs from one's own accent and that the listener has had little or no exposure to) is confounded with cognitive load. Note that such confounds also exist for other distortions of the speech signal, such as background noise. Listeners process speech in an unfamiliar accent slower and less efficiently (Floccia et al., 2006). It is thus unclear to which extent the network supporting accented speech perception is shared with the network associated with increased task/cognitive load processing. Notably, an increase in task difficulty/working memory load relates to increases in BOLD-activation in left insula (Wild et al., 2012), and in left MTG, SMA, left PTr, and right STG (Wild et al., 2012), and could therefore explain activations in these regions related to processing accented speech. Directly comparing the neural processing of familiar/unfamiliar accents may help distinguishing between the two networks.

## Accounts of accented and distorted speech processing

The current debate regarding how listeners understand others in challenging listening conditions focuses on the location and nature of neural substrates recruited for effective speech comprehension. The three accounts discussed below offer specific predictions regarding the neural networks involved in processing accented speech.

First, *auditory-only accounts* (Obleser and Eisner, 2009) hold that speech perception includes a prelexical abstraction process in which variation in the acoustic signal is “stripped away” to allow the perception system access to abstract linguistic representations. The abstraction process is placed at locations predominantly in the temporal (STS and STG) lobes. This account predicts that processing of accented speech takes place predominantly in the ventral stream, with minimal involvement of the dorsal stream.

Second, *motor recruitment accounts* suggest that auditory areas in the ventral stream and speech production areas in the dorsal stream are required to process unfamiliar speech signals (Wilson and Knoblich, 2005; Pickering and Garrod, 2013). These accounts assume that listening to speech results in the automatic activation of articulatory motor plans required for producing speech (Watkins et al., 2003). These motor plans provide forward models with information of articulatory mechanics, to be used when the incoming signal is ambiguous/unclear. Accented speech contains variation that can lead to ambiguities, and these accounts thus predict that perception of accented speech involves active involvement of speech production processes.

Third, *executive recruitment accounts* propose that activation of (pre-) motor areas during perception of distorted speech signals is not related to actual articulatory processing, but reflects the recruitment of general cognitive processes, such as increased attention, or decision processes (Rodríguez-Fornells et al., 2009; Venezia et al., 2012). Indeed, behavioral data suggest that recruitment of executive functions for processing accented speech (Adank and Janse, 2010; Janse and Adank, 2012; Banks et al., 2015) also predicts activation of frontal regions including left frontal operculum and anterior insula and precentral gyrus, as these regions have also been associated with executive functions such as working memory (Moisala et al., 2015).

The results in Table 1 contrast with predictions made by the auditory-only account (Obleser and Eisner, 2009), as areas associated with processing accent variation in Table 1 refer to a more widespread network than predicted. Instead, the network in Table 1 converges with the latter two accounts, as activation is located across ventral and prefrontal areas in the dorsal stream. We propose that these three accounts are synthesized into a single mixed account for processing of accented speech that brings together neural substrates associated with increased involvement of auditory and phonological processing (e.g., bilateral posterior STG), (pre-)motor recruitment for sensorimotor mapping (e.g., SMA), and substrates associated with increased reliance on cognitive control processes (e.g., IFG, insula, and frontal operculum).

## Concluding remarks

The neural mechanisms responsible for processing accent variation in speech are not clearly outlined, but constitute a topic of active investigation in the field of speech perception. However, to progress our understanding in this area, future studies should meet several aims to overcome previous design limitations.

First, experiments should be designed so that contributions from processing accented speech and effortful processing can be teased apart (Venezia et al., 2012). Second, studies should aim to distinguish between brain activity related to processing accent variation and other distortions, such as background noise. Adank et al. (2012a) contrasted sentences in a familiar accent embedded in background noise with sentences in an unfamiliar accent, to disentangle areas associated with processing accent-related variation from those associated with processing speech in background noise: Left posterior temporal areas in STG (extending to PT) and right STG (extending into insula) were more activated for accented speech than speech in noise, while bilateral FO/insula were more activated for speech in noise compared to accented speech, indicating that the neural architecture for processing accented speech and speech in background noise is not generic. Third, different accents vary in how much they deviate from the listener's own accent. Greater deviation between accents is associated with greater processing cost, but the neural response associated with variations in distance between accents has not been explored using fMRI. A recent study using Transcranial Magnetic Stimulation (TMS) showed a causal role for lip and tongue motor cortex in perceived speaker and listener distance processing (Bartoli et al., 2013). Another study used EEG to show that regional and foreign accents might be processed differently: processing sentences in an unfamiliar foreign accent reduces the size of the N400 compared to unfamiliar native accents (Goslin et al., 2012). It may be fruitful to use a wider variety of neuroscience techniques, including (combinations of) fMRI, EEG, MEG, and TMS, to investigate how the brain successfully accomplishes accented speech perception. Third, as processing effort, or cognitive load, is inevitably confounded with processing unfamiliar variation in accented speech, experiments should be designed to identify neural substrates associated with processing accent variation and those associated with increased cognitive load. One possibility would be to examine task difficulty and accent processing in a fully crossed factorial design to single out areas that show increased BOLD-activation for accented speech and for task difficulty. Finally, the contribution of production resources to processing accented speech should be examined, to explicitly test predictions from motor and executive recruitment accounts (e.g., Du et al., 2014).

## Conflict of interest statement

The authors declare that the research was conducted in the absence of any commercial or financial relationships that could be construed as a potential conflict of interest.
